# Tourette-like behaviors in the normal population are associated with hyperactive/impulsive ADHD-like behaviors but do not relate to deficits in conditioned inhibition or response inhibition

**DOI:** 10.3389/fpsyg.2014.00946

**Published:** 2014-09-02

**Authors:** Nadja Heym, Ebrahim Kantini, Hannah L. R. Checkley, Helen J. Cassaday

**Affiliations:** ^1^School of Psychology, University of NottinghamNottingham, UK; ^2^Division of Psychology, School of Social Science, Nottingham Trent UniversityNottingham, UK

**Keywords:** Attention-Deficit/Hyperactivity Disorder, Tourette Syndrome, conditioned inhibition, automatic inhibition, executive inhibition

## Abstract

Attention-Deficit Hyperactivity Disorder (ADHD) and Tourette Syndrome (TS) present as distinct conditions clinically; however, comorbidity and inhibitory control deficits have been proposed for both. Whilst such deficits have been studied widely within clinical populations, findings are mixed—partly due to comorbidity and/or medication effects—and studies have rarely distinguished between subtypes of the disorders. Studies in the general population are sparse. Using a continuity approach, the present study examined (i) the relationships between inattentive and hyperactive/impulsive aspects of ADHD and TS-like behaviors in the general population, and (ii) their unique associations with automatic and executive inhibitory control, as well as (iii) yawning (a proposed behavioral model of TS). One hundred and thirty-eight participants completed self-report measures for ADHD and TS-like behaviors as well as yawning, and a conditioned inhibition task to assess automatic inhibition. A sub-sample of fifty-four participants completed three executive inhibition tasks. An exploratory factor analysis of the TS behavior checklist supported a distinction between phonic and motor like pure TS behaviors. Whilst hyperactive/impulsive aspects of ADHD were associated with increased pure and compulsive TS-like behaviors, inattention in isolation was related to reduced obsessive-compulsive TS-like behaviors. TS-like behaviors were associated with yawning during situations of inactivity, and specifically motor TS was related to yawning during stress. Phonic TS and inattention aspects of ADHD were associated with yawning during concentration/activity. Whilst executive interference control deficits were linked to hyperactive/impulsive ADHD-like behaviors, this was not the case for inattentive ADHD or TS-like behaviors, which instead related to increased performance on some measures. No associations were observed for automatic conditioned inhibition.

## Introduction

Attention-Deficit/Hyperactivity Disorder (ADHD) and Tourette Syndrome (TS) present as distinct conditions clinically; however, there is evidence of comorbidity between those two disorders and inhibitory control deficits have been proposed for both (Eddy et al., 2009). ADHD is a neurodevelopmental disorder characterized by excessive inattention, hyperactivity and impulsivity, and diagnosis typically comprises three subtypes for inattentive, hyperactive/impulsive, or combined ADHD cases. ADHD has predominantly been studied using clinical populations of children and/or adults, and this research suggests that the psychological symptoms of ADHD derive from deficient inhibitory control (Barkley, 1997; Quay, 1997; Ozonoff et al., 1998). As the symptoms and behaviors have been suggested to be dimensional with extreme manifestations leading to diagnosis (Coghill and Sonuga-Barke, 2012), research has also begun to examine individual differences in ADHD-like behaviors and their links to response inhibition in the general population (Kuntsi et al., 2005; Herrmann et al., 2009).

TS is also a neurodevelopmental hyperkinetic disorder, in this case involving sudden, repetitive unintentional movement-based tics (motor tics) and involuntary sounds or utterances produced by moving air through the nose, mouth, or throat (phonic tics). Phonic and motor tics relate to separate diagnostic criteria as they involve discrete muscle groups, and phonic tics are generally more common (Leckman et al., 2006). Factor analytic studies (Khalifa and Knorring, 2005; Robertson et al., 2008) have shown that there are pure or uncomplicated (without comorbidity, prevalent in 10% of patients) and comorbid phenotypic expressions of TS, mainly associated with ADHD and obsessive-compulsive disorder (OCD) (Bloch and Leckman, 2009; Cavanna et al., 2009). Consequently, many clinical studies include patients with symptoms of ADHD or OCD. Attentional and impulse control problems are thought to precede the emergence of TS symptoms, and it is the behavioral disturbances and impaired executive functioning typical of ADHD that appear to be most closely linked to TS (for review see Robertson, 2000). The obsessive-compulsive behaviors (OCBs) most characteristic of TS appear to be clinically different from those seen in pure OCD, and involve repetitive thoughts of aversive content, and compulsions to do with checking, sorting and arranging (Robertson, 2000). TS has been linked to cognitive and executive functioning impairment, however, the specificity of these deficits to pure TS as opposed to comorbid conditions is less clear (Eddy et al., 2009). Moreover, as TS-like behaviors involve separate behavioral aspects (phonic and motor), these may show differential associations with the subtypes of ADHD, OCBs, and response inhibition deficits. Whilst research has begun to examine individual differences in ADHD-like behaviors in the general population, this is not the case for TS. However, it is recognized that TS lies at the extreme of what can be viewed as a tic disorder spectrum which includes also “transient,” “chronic,” and “non-specific” tic disorders (Leckman, 2002). Therefore, the main aim of this study is to examine individual differences in the general population in the expression of behaviors similar to those seen in TS and ADHD and their links to response inhibition.

Inhibition is a broad but nonetheless useful construct, particularly in relation to the deficits characteristic of a range of psychopathologies. Based on an extensive review, Nigg (2000) proposed an integrated taxonomy of inhibition in which inhibitory control (broadly defined) includes executive, automatic and motivational inhibitory processes, each corresponding to separate cognitive, personality and neural underpinnings. Differentiating amongst these allows more systematic identification of the specific inhibitory control mechanisms linked to various psychopathologies (Nigg, 2000). In the case of ADHD and TS, the most widely studied inhibitory processes fall under the class of effortful executive inhibition. This includes interference control of motor or cognitive responses due to resource or stimulus competition—typically assessed by tasks which require respondents to suppress (their perception of) a stimulus and competing response in order to execute the primary response, such as in the standard Stroop or Flanker tasks. Similarly, behavioral inhibition requires suppressing a dominant or pre-potent automatic response option—typically assessed with the Go/No-Go or Stop-Signal tasks. A variety of studies using these tasks, have demonstrated deficits in executive response inhibition in children and adults with ADHD (Schachar and Logan, 1990; Iaboni et al., 1995; Seidman et al., 1997; Konrad et al., 2000; Schachar et al., 2000; Young et al., 2006). A meta-analysis suggested slower go and stop reaction times in ADHD children; however, this was non-specific in that children with conduct disorder showed similar deficits (Oosterlaan et al., 1998). Importantly, though a number of studies report executive inhibition deficits in ADHD, comorbidity was not accounted for in most, and those studies that did, typically found that deficits are not necessarily specific to ADHD. Indeed, a more recent meta-analysis of executive dysfunction in clinic-referred and community ADHD samples (across 83 studies) found only moderate effect sizes, consistent with the lack of universality of these deficits (Willcutt et al., 2005).

Similar to ADHD, it has been argued that TS may be a result of an inhibitory dysfunction (Sheppard et al., 1999). While some studies have found inhibitory deficits (Georgiou et al., 1995; Marsh et al., 2004; Crawford et al., 2005), the overall evidence of inhibitory impairment in TS is inconsistent, and has similarly been attributed to comorbidity issues (Pennington and Ozonoff, 1996). Indeed, a number of studies report no significant difference in cognitive and behavioral executive response inhibition between TS groups without comorbid ADHD and matched controls. For example, participants with pure TS showed no performance deficits on Go/No-Go (Serrien et al., 2005; Roessner et al., 2008), color-word Stroop or Flanker tasks (Channon et al., 2003, 2006, 2009). Similarly, Ozonoff et al. (1998) found normal inhibition effects in children with mild TS but impaired inhibition in children with TS and comorbid ADHD or OCD. Thus, it has been suggested that ADHD comorbidity may contribute to, or possibly be responsible for the inhibitory deficits observed in TS (Ozonoff et al., 1998; Como, 2001; Channon et al., 2003; Gilbert et al., 2004; Eddy et al., 2009). Indeed, Jackson and colleagues have shown that, despite their general difficulties with inhibition, pure TS participants (without comorbidity) show paradoxically enhanced volitional control in suppressing established learned associations, in both saccadic and manual switching tasks (Mueller et al., 2006; Jackson et al., 2007, 2011a; Jung et al., 2014). Such tasks rely on executive processes to show the required flexibility when the response requirement is changed. Thus, the above studies of inhibitory (dys)function have used volitional response measures involving conscious control, which can be classified as effortful executive inhibition category (Nigg, 2000). To date, little research has examined motivational or automatic inhibitory processes in ADHD and TS, though clinical studies suggest that automatic attentional inhibition may be of particular importance in the inattentive subtype of ADHD (Aman et al., 1998). To gain specificity regarding unique deficits in different psychopathologies, the ideal approach is to simultaneously examine and distinguish different types of response inhibition deficits using several response inhibition tasks and in more than one disorder, whilst controlling for comorbidity (Nigg, 2000).

From a learning theory perspective, successful performance on such tasks detailed above requires inhibition of pre-potent stimulus-response (S-R) associations. The learning of stimulus-stimulus (S-S) associations follows the general laws of associative learning and may well rely on similar mechanisms, however, there are differences in the specific neural circuitries involved depending on the type of S-R (Jog et al., 1999; Killcross and Coutureau, 2003; Yin and Knowlton, 2006) or S-S conditioning procedure in use (Daum et al., 1993; Fanselow and Poulos, 2005; Kim and Jung, 2006). Given that different neural circuitries are necessary for S-R and S-S associations, we cannot assume that both types of learning are affected in ADHD or TS. Indeed, when performance on procedural (S-R) learning tasks was systematically compared with tasks requiring associative learning (based on S-S as well as S-R associations) in TS patients, the underlying learning systems were suggested to be dissociable (Marsh et al., 2005). However, there have been few reported studies of S-S learning and the role of automatic inhibition in ADHD or TS. Studies using inhibition of return have found no evidence for such inhibitory deficits in cases of TS without comorbidity (Yuen et al., 2005). Similarly, negative priming, though impaired in OCD, appears to be spared in TS and adult ADHD (Ozonoff et al., 1998; Nigg et al., 2002). A latent inhibition study—in which stimulus pre-exposure attenuates later S-S learning—found also no deficit in TS participants (Swerdlow et al., 1996). However, although latent inhibition procedures effectively inhibit the acquisition of a new association, they do not render the pre-exposed stimulus truly inhibitory (Baker and Mackintosh, 1977). True inhibition is demonstrated by establishing a stimulus selectively to predict the occasions on which an otherwise expected outcome will not occur (Pavlov, 1927; Rescorla, 1969), as seen in conditioned inhibition (CI) procedures. Thus, the inhibition of S-S associations (termed conditioned inhibition) has been defined in terms of the learned ability of a stimulus to inhibit an earlier established association (Rescorla, 1969). More specifically, the presence (during an excitatory association) of a stimulus which signals the absence of the otherwise expected event, establishes the additional stimulus as inhibitory (Pavlov, 1927). Since CI can be established in both implicit and explicit learning variants, it should be classified as automatic rather than effortful inhibition.

CI has only recently been examined in clinically diagnosed children and adolescents with ADHD (Kantini et al., 2011a) and TS in the absence of comorbid ADHD (Kantini et al., 2011b). Although there was no evidence for any differences in CI between ADHD or TS groups compared to matched controls, in both disorders performance was dependent on medication. In ADHD participants, both higher dosage and longer duration of treatment with methylphenidate were related to improved CI when symptom severity was taken into account (Kantini et al., 2011a). On the other hand, medication with clonidine for TS impaired CI (Kantini et al., 2011b). Thus, differences in CI in ADHD and TS were related to medication rather than diagnosis. Hence, previous studies conducted in clinically diagnosed and treated populations may be limited to the extent that observed performance differences may be confounded by medication status as well as comorbidity. Moreover, given the difficulties in recuiting pure cases (without comorbidity), the majority of experimental studies conducted in clinical populations have looked at ADHD without differentiating between hyperactive/impulsive and inattentive subtypes of the disorder.

Establishing differential performance deficits based on behavioral subtypes of either ADHD or TS may be also key to the delineation of their respective deficits. To date a relatively small number of studies have addressed the relationship between inhibition deficits and subtypes of ADHD. Willcutt et al.'s (2005) meta-analysis in clinical samples supports executive performance deficits in combined and inattentive ADHD subtypes, whereas impairment in the hyperactive/impulsive subtype was minimal (though only three studies included the latter). Herrmann et al. (2009) found executive response inhibition deficits for inattentive but not hyperactive/impulsive ADHD-like behaviors in a healthy non-clinical adult sample. Thus, the subtypes of ADHD appear to be differentially related to response inhibition deficits, but further evidence is needed. Whilst recent studies began to examine executive response inhibition deficits linked to subtypes of ADHD-like behaviors in the clinical and general population, to date there are no studies that have been applied to TS-like behaviors in a similar way. Moreover, whilst automatic attentional inhibition has been suggested to be of particular importance in the inattentive subtype of ADHD (Aman et al., 1998), there are no studies that have examined the association of the subtypes of ADHD with automatic inhibition deficits in the general population.

Therefore, following a dimensional approach, the main aim of the current study was to examine (i) the associations between the different behavioral aspects of both conditions in the general population, and (ii) their unique roles in inhibitory control deficits, specifically in automatic inhibition. Few studies have used various tasks of response inhibition simultaneously to assess disorder and subtype specificity of deficits (Nigg, 2000), and indeed, none has explicitly compared performance on standard response inhibition tasks and CI of S-S associations in the normal population. Therefore, extending the experimental approaches previously adopted in the ADHD and TS literature, the current study set out to examine automatic response inhibition deficits related to the subtypes of TS- and ADHD-like behaviors in the general population and compare those to executive response deficits in a subsample.

Whilst there are established ADHD scales for use in normal populations (ASRS; Kessler et al., 2005), that differentiate between hyperactive/impulsive and inattentive behaviors, currently there are no such scales to measure TS-like behaviors in normal populations. Therefore, we developed a short behavioral checklist similar in format to the ASRS tapping into the different behavioral aspects of TS (including pure motor and phonic tic related behaviors, as well as OCBs) in order to examine the overlap between TS-like behaviors with ADHD subtypes and their associations with automatic and effortful response inhibition deficits. Moreover, tics can be triggered by various situations and are often preceded by premonitory sensations (Prado et al., 2008), which may become tic-generating stimuli through S-S associations (Robertson, 2000). For example, whilst tics can be temporarily suppressed using distraction, they appear to increase during stress and relaxation after stress (Jankovic, 1997). Yawning is a stereotyped repetitive motor act occurring during such situations, increasing arousal and self-awareness, and it has been suggested that excessive yawning is associated with and triggering TS tics (Dalsgaard et al., 2001; Walusinski et al., 2010). Both, yawning and TS (like a number of other bodily sensations) have been conceptualized as “urges for action,” and based on the overlap in the functional anatomy, yawning has been proposed as a behavioral model for TS (Jackson et al., 2011b). It has been suggested that identifying premonitory sensations and subjective experiences associated with symptom expression may be useful in identifying more homogenous subgroups of TS (Prado et al., 2008). Therefore, we also included a scale assessing yawning in different contexts (Greco and Baenninger, 1993) to examine its associations with different TS-like behaviors.

The hypotheses tested were as follows: (i) based on comorbidity in clinical groups, ADHD- and TS-like behaviors were predicted to be positively associated; (ii) in line with the notion of the behavioral yawning model for TS, TS-like behaviors were predicted to be associated with excessive yawning also in contexts unrelated to fatigue or boredom; (iii) automatic attentional inhibition deficits were expected only in relation to the inattentive ADHD subscale (Aman et al., 1998); (iv) controlling for “comorbidity,” ADHD-like behaviors were predicted to be more strongly associated with deficits in tasks measuring executive inhibitory control; whereas any apparent relationships with TS-like behaviors are due to ADHD-like behaviors or OCBs known to co-occur with TS.

## Materials and methods

### Participants

The full sample consisted of 138 mixed undergraduate and community participants (90 females, 48 males) with a mean age of 23.54 (*SD* = 4.62; 17–40 years), who completed all psychometric scales and the CI task. A subsample (*N* = 54; 34 females and 20 males; mean age of 22.98, *SD* = 4.71), recruited and tested under the same conditions, took also part in the executive behavioral inhibition response tasks. (There were no significant differences in any of the individual difference measures between the subsample completing the additional tasks and those who did not (*t*s < 1.53, *p*s n.s.). There were also no significant sex differences for all variables (*t*s < 1.63, *p*s n.s.) apart from Yawning-active (*t* = −2.19, *p* = 0.04) with females yawning more than males during activity).

### Ethics statement

The study was approved by the School of Psychology Research Ethics Committee of the University of Nottingham, and the R and D Departments of the Nottinghamshire Lincolnshire Partnership NHS Trust (Derbyshire REC, ref 08/H0401/34, approved April 2008). After complete description of the study, written consent was acquired from all participants (or written consent from parents and verbal assent from minor participants) prior participation.

### Measures and materials

#### Psychometric scales

ADHD-like behaviors were measured using the 18-item Adult ADHD Self-Report Scale (ASRS; Kessler et al., 2005). The scale excluded any diagnostic referential items to be suitable to use within normal non-diagnosed populations. Questions referred to the frequency occurrence of ADHD-like behaviors over the past 6 months, and participants responded on a 5-point scale (0 = never, 1 = rarely, 2 = sometimes, 3 = often, and 4 = very often). The ASRS can be split into two scales: ASRS-inattention and ASRS-hyperactivity/impulsivity (consisting of 9 items each). Previous alphas for the scales were found to be in the range of 0.63–0.72 (Kessler et al., 2005).

Tourette-like behaviors were assessed using an in-house 18-item behavior checklist based on the same format as the aforementioned ASRS. The items were developed to tap into most of the DSM-IV and ICD10 symptoms for TS (except common complex tics as these would be unlikely in an undiagnosed population). Items were framed to assess variation in symptomatic behaviors within the normal range, and referred to frequency occurrences of both TS-like phonic and motor symptomatology including unintended oral sounds (e.g., throat clearing, coughing, swearing), facial and body related motor behaviors (e.g., eye blinking, shrugging, head movements). We also included three items referring to TS related OCBs (Robertson, 2000) given the co-occurrence of OCD in TS (Swerdlow, 2001; Rankins et al., 2005; Thibault et al., 2008). In line with the specific clinical distinction of TS-related OCBs, these items referred to repetitive thoughts (without giving a specific theme or content), checking and ordering behaviors, and were worded similarly to the ASRS and the rest of the TS items. Participants were required to indicate frequency of symptom occurrence on a 5-point scale (0 = never, 1 = rarely, 2 = sometimes, 3 = often and 4 = very often). As phonic and motor tics involve discrete muscle groups and phonic tics are generally more common (Leckman et al., 2006), we conducted a principal component analysis on the fifteen pure TS items to identify an underlying factor structure. The three OCB items were analyzed separately (in order to examine their association with pure TS-like behaviors), but were also included in the overall TS scale to reflect OCB co-occurrence.

Yawning in different contexts was assessed using the 24-item Yawning (YWN) scale (Greco and Baenninger, 1993). Participants were required to indicate how likely they would be to yawn in various everyday situations (e.g., when in a lecture, giving a speech, after a meal) and at different times of the day (e.g., morning, evening) on a 6-point scale (0 = not at all, 1 = just a little, 2 = somewhat, 3 = moderately, 4 = quite a lot, 5 = very much). Again, a principal component analysis was conducted to identify the underlying factor structure of those items.

#### Experimental tasks

The tasks were run using E-Prime (Psychology Software Tools Inc., Pittsburgh, USA) on a standard 17” monitor computer. The Go/No-Go, Simon and Stroop tasks were administered using a standard keyboard (for one set of participants) as well as a Cedrus RB-730 response pad (for another set of participants) to examine whether use of a colored response pad would produce faster responding instead of colored keyboard buttons, and eliminating any potential keyboard lags (button pressing vs. response recording). However, no reaction time differences between responding using the keyboard or response pad were found and as such the samples were combined. The CI task used the standard computer keyboard as response input device.

***Conditioned inhibition task***. Inhibitory learning was measured using the “Mission to Mars” task (Kantini et al., 2011a,b)—a modified version of an established CI paradigm (Migo et al., 2006). Specifically, the task was modified to be more engaging: it used a hypothetical scenario of a fleet of star ships traveling on an exploration to Mars, and participants were required to learn which processes (stimulus associations) led to success or failure (survival vs. explosion of their fleet). The length of the original task was likely to be too taxing on the maintenance of attention, particularly in relation to (sub-) clinical ADHD. Therefore, the number of training and test trials was reduced providing a sufficient number of trials to leave the acquisition of CI unaffected. The training phase consisted of 45 learning trials with nine cycles of five stimulus sequences. The test phase followed immediately, and consisted of 20 trials with five cycles of four stimulus sequences in which the novel generalized stimulus (Sg) was introduced. Sequences were presented in a random order within each cycle in both phases. The procedure for test trials was identical to the training, except that prior to the presentation of the unconditioned stimulus (UCS) or its absence, participants were presented with an on-screen rating scale requiring participants to estimate the likelihood of success in the task on a 9-point scale (1 = highest chance of failure, 5 = uncertainty, 9 = highest chance of success). An expectancy survival score of 1 showed maximum inhibitory learning when presented with the inhibitor, whereas a score of 9 showed maximum excitatory learning when the inhibitor was absent. In this task summation tests measure the generalization of the inhibitory properties of the conditioned inhibitor to (i) Sg which does not appear at all in the training phase but is sufficiently similar to produce generalized responding; and (ii) a transfer conditioned stimulus (CSt) that does not appear with the inhibitor in the training phase. As was the case for the clinical studies of TS and ADHD (Kantini et al., 2011a,b), the task used serial presentation of the CI followed by CSt or Sg. CI is demonstrated when reinforced stimuli receive higher expectancy ratings than non-reinforced stimuli presented with the pre-trained CI. The CI ratio score was calculated by dividing the average expectancy scores for trials with the inhibitor by the average expectancy scores of the non-inhibited trials. Thus, a smaller CI ratio score indicates better CI.

***Stroop task***. Interference control was assessed using a computerized version of the classic color-word Stroop task (Stroop, 1935). Participants were shown color words (“blue,” “red,” or “green”), which were displayed either in blue, red or green ink. They were required to respond to the ink color not the color word by pressing corresponding buttons (4 = green, 5 = red, 6 = blue). There were two conditions: (i) congruent, where the color word matched the ink color; and (ii) incongruent where the color word was different than the ink color. The practice session consisted of 12 randomly presented trials (6 congruent, 6 incongruent) and the testing session of 48 trials (24 congruent, 24 incongruent). Participants received accuracy feedback during the practice, but not testing session. Reaction times and accuracy for congruent and incongruent trials were recorded. Stroop interference effects were calculated by subtracting reaction times for congruent trials from incongruent trials.

***Go/No-Go task***. Behavioral inhibition of a pre-potent response was assessed using the Go/No-Go task (obtained from The Sackler Institute for Developmental Psychobiology, 2008). Participants were required to respond as quickly as possible to a Go signal (mole) by pressing the “spacebar” on the keyboard, but holding back responses to No-Go signals (vegetables). The training session was composed of seven Go and three No-Go trials, followed by the testing session composed of four blocks with an average total of 55 trials per block resulting in an average of 41 go and 14 no-go trials. Trials were presented in random order within each block. Overall accuracy and reaction times for Go trials were recorded.

***Simon task***. Response selection interference was assessed using an adapted version of the Simon task (Simon and Rudell, 1967; Simon, 1969), which measures response interference from an independent (indirect unconditional) parallel processing path on the direct conditioned response in terms of a spatial inference on the primary perception response. The task required participants to correctly and as quickly as possible respond to the color of displayed squares by pressing the left key (for green) or the right key (for red) but only after a Go signal appeared (box around the fixation cross). Targets appeared on the left or right of the fixation cross, and the location of the red or green squares was counterbalanced such that the color targets were either presented congruently to the location of the colored response key and the responding hand (green on left side, red on right side) or incongruently (green on right side, red on left side). Performance is normally more accurate and faster when the perceptual stimulus-response associations share the same relative spatial location. Participants were instructed to ignore the location of the target and respond only to the color shown. The Go signal was presented either following a 100 ms or a 900 ms delay. Participants could only respond once the Go signal was presented, hence a longer delay should lead to fewer errors and subsequently faster response times because they have had more time to process the stimuli and select the correct response option. The practice session consisted of eight trials and the test session of 160 trials (40 congruent and 40 incongruent at 900 ms delay, and 40 congruent and 40 incongruent at 100 ms delay). Average response time scores were computed for the 100 and 900 ms delay conditions. As each trial continued until the participant produced the correct response, response times serve as an indirect measure of response accuracy.

### Procedure

All 138 participants were subject to the CI task followed by the questionnaires for ADHD- and Tourette-like behaviors and yawning. The subsample of 54 participants completed in addition the three executive behavioral response tasks: the Stroop, Simon and Go/No-Go tasks.

### Statistical analyses

Two separate principal component analyses with varimax rotation were conducted to examine the underlying factor structures of the pure TS-like behavioral items and the Yawning scale (YWN) items. Items with loadings <0.30 and /or strong cross-loadings (difference between loadings <0.10 or cross-loadings on more than 2 factors) were excluded from the subscales. Scale scores were calculated for the overall scales (including all items) and the subscales by summing the responses of the respective items. Pearson correlations were used to examine the zero-order associations between the ASRS, TS, and YWN scales. To further assess the unique contributions of the ASRS subscales on the TS subscales, and the ASRS and TS subscales on yawning behaviors, multiple regression analyses were conducted. To account for associations between ADHD and TS scales, partial correlations were computed for the relationships of ASRS and TS scales (controlling for each other) with the behavioral measures. Due to the interrelationships between variables, correlations were corrected using the false discovery rates procedure (Benjamini and Hochberg, 1995), rather than Bonferroni which has less statistical power (Perneger, 1998; Nakagawa, 2004). Thus, uncorrected significant *p*-values are indicated in the tables.

## Results

### Exploratory factor analyses of TS and YWN scale items

For the pure TS items, parallel analysis indicated a two factor structure accounting for 35.85% of the variance (*KMO* = 0.77; Bartlett's test *p* < 0.001, see Table 1 for full item content and TS items factor loadings). Eight items loaded on factor one labeled TS-phonic and included questions related to sounds produced through the nose, mouth, or throat (e.g., “*sniff when not ill*”). The remaining seven items loaded onto factor two labeled TS-motor, and included questions related to unintentional physical movements (e.g., “*make unintentional movements*,” “*randomly shrug shoulders*”). There were no cross-loading items. Four summed scale scores were calculated—one overall scale score for all TS items including the three OCB items (TS-overall) and three subscales scores (TS-phonic, TS-motor, TS-OCB), such that higher scores on each scale indicates greater frequency of endorsement of the respective behaviors, respectively. The two pure TS-factors were positively correlated with each other (*r* = 0.522, *p* < 0.001), and TS-OCB was positively correlated with both TS-phonic (*r* = 0.216, *p* < 0.05) and TS-motor (*r* = 0.276, *p* < 0.01). To test the difference between the dependent correlation coefficients, *t*-tests showed that the correlation between phonic and motor TS is significantly larger than the correlations of TS-OCB with phonic (*t* = 3.14, *df* = 135, *p* < 0.001) and motor TS (*t* = 2.65, *df* = 135, *p* < 0.01).

**Table 1:** **Factor loadings of pure TS items**.

**Items (*How often …*)**	**TS-phonic**	**TS-motor**
9. do you cough when not ill	0.756	
12. do you sniff when not ill	0.742	
13. do you repeatedly clear throat for no reason	0.602	
1. do you yawn when not tired	0.493	
17. do you say things you wish you hadn't	0.468	
7. do you swear without provocation	0.417	
2. do you bite lips or cheeks	0.357	
16. do you make unintentional sounds	0.345	
5. does your head move in a way you did not intend		0.725
4. do you randomly shrug your shoulders		0.706
10. do you experience rapid blinking		0.681
8. do you grunt without reason	0.378	0.569
11. do you have uncontrollable face twitches		0.482
14. do you make unintentional movements		0.427
18. do you shout for no reason		0.423
Eigenvalues	3.98	1.39
***Additional OCB items:***		
3. do you tidy things into fixed order/arrangement		
6. do you check electrical things are switched off		
15. are you bothered by repetitive unpleasant images in your mind		

*Factor loadings >0.30 are displayed*.

For the YWN scale, parallel analysis indicated a three factor structure accounting for 47.48% of the variance (*KMO* = 0.80, Bartlett's test *p* < 0.001, see Table 2 for factor loadings). Ten items loaded onto factor one labeled YWN-active and comprised of situations involving activity and concentration (e.g., “*working*”). Seven items loaded onto factor two labeled YWN-inactive and comprised of situations involving inactivity or waiting (e.g., “*sitting in traffic jam*”). Five items loaded on the third factor labeled YWN-stress and consisted of stressful situations involving self-presentation or evaluation (e.g., “*giving a speech or lecture*”). Two cross-loading generic items were excluded (“*lack of sleep*” and “*tired*”). Four summed scales were calculated—one overall scale score including all items (YWN-overall) and the three factor scales scores (YWN-active, YWN-inactive, YWN-stress), such that higher scores reflected a greater tendency to yawn during the respective situations. YWN-inactive was significantly positively correlated with YWN-active (*r* = 0.564, *p* < 0.001) and YWN-stress (*r* = 0.237, *p* < 0.01), whereas YWN-active and YWN-stress were not significantly correlated (*r* = 0.157, *p* = 0.07).

**Table 2:** **Factor loadings of YWN scale items**.

**Items (*How often do you yawn when…*)**	**YWN-active**	**YWN-inactive**	**YWN-stress**
21. working	0.733	0.302	
16. lecture/ Class	0.722		
20. see others yawn	0.680		
1. listening to speech or lecture	0.625		
11. morning	0.621		0.367
10. getting out of bed in morning	0.597		
23. in church	0.573		
22. study at night	0.570	0.319	
4. driving at night on lonely road	0.475		
24. while taking this survey	0.466		
19. after a meal		0.715	
2. sitting in a traffic jam		0.646	
17. lack of sleep^a^	0.307	0.622	−0.369
13. evening		0.586	
12. afternoon	0.385	0.574	
3. waiting for train/bus		0.573	
5. driving on sunny day (no traffic)		0.551	
15. tired^a^	0.451	0.535	−0.376
9. lying in bed before going to sleep		0.413	
8. being interviewed for job			0.824
14. on dates			0.757
7. waiting to begin competitive event			0.717
6. giving speech or lecture			0.648
18. stressed		0.332	0.496
Eigenvalue	6.50	3.22	1.68

Factor loadings >0.30 are displayed; superscript

a *denotes excluded items*.

### Descriptive statistics and zero-order correlations between ASRS, TS and YWN scales

The means, standard deviations (SD), Cronbach's alphas or mean inter-item correlations for scales with less than 5 items (MICs greater than 0.30 are deemed reliable; Robinson et al., 1991), and zero-order correlations between scales are shown in Table 3. The Cronbach's alphas for all scales were good (>0.70), apart from TS-phonic which was slightly lower but still acceptable (α = 0.69), whereas TS-OCB showed low internal consistency (α = 0.56; *MIC* = 0.246). TS-OCD and YWN-stress were positively skewed and transformed as appropriate to normalize the scales. TS phonic and motor (and overall), were positively correlated with the three YWN subscales (and overall), as well as both ASRS subscales (and overall), whereas TS-OCB was positively correlated only with YWN-inactivity (and overall) and ASRS hyperactivity/impulsivity (but not overall). All ASRS scales were significantly positively correlated with YWN-activity (and overall), and ASRS hyperactivity/impulsivity was also positively correlated with YWN-inactivity. The ASRS scales were not associated with YWN-stress.

**Table 3:** **Descriptive statistics and correlations for ASRS, TS and YWN scales**.

**Scales**	**Descriptive statistics**			**Correlations**									
	**Alpha (MIC)**	**Mean**	**SD**	**ASRS-overall**	**ASRS-hyper/ imp**.	**ASRS-inattent**.	**TS-overall**	**TS-phonic**	**TS-motor**	**TS-OCB**	**YWN-overall**	**YWN-active**	**YWN-inactive**
ASRS-overall	0.81	32.13	8.16	1									
ASRS-hyper/imp.	0.73	14.73	4.72	0.866^***^	1								
ASRS-inattent.	0.72	17.40	4.71	0.865^***^	0.499^***^	1							
TS-overall	0.78	22.82	7.73	0.425^***^	0.476^***^	0.259^**^	1						
TS-phonic	0.68	11.48	4.17	0.469^***^	0.483^***^	0.329^***^	0.845^***^	1					
TS-motor	0.72	6.32	3.57	0.328^***^	0.332^**^	0.235^**^	0.821^***^	0.522^***^	1				
TS-OCB ^t^	(0.246)	2.40	0.49	0.060	0.191^*^	−0.087	0.546^***^	0.216^*^	0.267^**^	1			
YWN-overall	0.87	46.72	15.76	0.359^***^	0.333^***^	0.289^**^	0.529^***^	0.485^***^	0.409^***^	0.240^**^	1		
YWN-active	0.84	23.36	9.14	0.431^***^	0.367^***^	0.378^***^	0.444^***^	0.471^***^	0.305^***^	0.141	0.893^***^	1	
YWN-inactive	0.76	12.67	5.99	0.175^*^	0.221^**^	0.082	0.494^***^	0.379^***^	0.399^***^	0.336^***^	0.827^***^	0.564^***^	1
YWN-stress ^t^	0.75	0.49	0.35	0.149	0.153	0.105	0.218^**^	0.167^*^	0.250^**^	0.051	0.386^***^	0.157	0.237^**^

N = 138;

* *p < 0.05*,

** *p < 0.01*,

*** *p < 0.001 (FDR adjusted; Benjamini and Hochberg, 1995); superscript t denotes transformed variable; MIC = mean inter-item correlation for scales with less than five items*.

### Regression analyses for ASRS subscales associations with TS subscale behaviors

To examine the unique associations of the ASRS subscales with the TS subscales, three linear regression analyses were conducted. For TS-phonic, the overall model was significant (*R* = 0.50, *R*^2^ = 0.24, *F* = 21.75, *p* < 0.001); ASRS-hyperactivity/impulsivity (β = 0.424, *t* = 4.91, *p* < 0.001) was significantly associated with TS-phonic, whereas ASRS-inattention did not reach significance (β = 0.117, *t* = 1.36, *p* = 0.18). For TS-motor, the overall model was significant (*R* = 0.34, *R*^2^ = 0.12, *F* = 8.60, *p* < 0.001); again, only ASRS-hyperactivity/impulsivity (β = 0.285, *t* = 3.06, *p* < 0.01) was significantly associated with TS-motor, whereas ASRS-inattention was not (β = 0.092, *t* = 0.99, *p* = 0.32). For TS-OCB, the overall model was significant (*R* = 0.28, *R*^2^ = 0.08, *F* = 5.89, *p* < 0.001); interestingly, whilst both subscales were significantly associated with TS-OCB, ASRS-hyperactivity/impulsivity (β = 0.311, *t* = 3.27, *p* = 0.001) was associated with an increase whereas ASRS-inattention (β = −0.242, *t* = −2.54, *p* < 0.05) with a decrease in TS-OCB.

### Regression analyses for ASRS and TS subscales associations with YWN

The unique associations of the ASRS and TS subscales with the YWN subscales were examined with three linear regression analyses. For YWN-active, the overall model was significant (*R* = 0.54, *R*^2^ = 0.29, *F* = 10.70, *p* < 0.001); only ASRS-inattention (β = 0.234, *t* = 2.65, *p* < 0.01) and TS-phonic (β = 0.327, *t* = 3.48, *p* = 0.001) were significantly associated with YWN-active but not ASRS-hyperactivity/impulsivity, TS-motor or OCB (*p*s > 0.40). For YWN-inactive, the overall model was significant (*R* = 0.50, *R*^2^ = 0.25, *F* = 8.72, *p* < 0.001); only the TS scales, TS-phonic (β = 0.214, *t* = 2.21, *p* < 0.05), TS-motor (β = 0.231, *t* = 2.54, *p* < 0.05) and TS-OCB (β = 0.223, *t* = 2.74, *p* < 0.01) were significantly associated with YWN-inactive but not the ASRS scales (*p*s > 0.73). For YWN-stress, the overall model was non-significant (*R* = 0.26, *R*^2^ = 0.07, *F* = 1.95, *p* = 0.09); and TS-motor was the only “predictor” that reached significance (β = 0.220, *t* = 2.18, *p* = 0.03; for all other scales *p*s > 0.51).

### Partial correlations of ASRS and TS scales with behavioral measures

The partial correlations between the ASRS and TS scales with the behavioral tasks are shown in Table 4. Contrary to hypothesis (iii), none of the scales were associated with CI; however, there were some associations with the executive inhibition tasks. ASRS-inattention was significantly associated with reduced Stroop reaction times for congruent and incongruent trials and interference, whereas ASRS-hyperactivity/impulsivity was associated with increased reaction times for the Simon effect at 900 ms. TS-phonic and motor were not associated with any of the behavioral measures, though overall TS was associated with increased Stroop reaction times for congruent trials and increased Stroop accuracy. TS-OCB was linked to increased accuracy in the Go/No-Go task.

**Table 4:** **Partial correlations of ASRS and TS scales with behavioral data**.

**Behavioral scores**	**ASRS-overall**	**ASRS-hyper/impulsivity**	**ASRS-inattention**	**TS-overall**	**TS-phonic**	**TS-motor**	**TS-OCB**
CI ratio	−0.028	−0.007	−0.035	0.029	0.092	−0.075	0.011
Stroop ACC	−0.189	−0.091	−0.144	0.273^*^	−0.035	0.201	0.177
Stroop RT congruent	−0.289^*^	0.023	−0.343^*^	0.350^**^	0.125	0.135	0.145
Stroop RT incongruent	−0.260	0.176	−0.451^***^	0.237	0.040	0.128	0.033
Stroop RT interference	−0.073	0.270	−0.355^*^	−0.064	−0.104	0.038	−0.142
Go/No-Go ACC	−0.180	−0.182	0.012	0.100	−0.020	−0.066	0.297^*^
Go/No-Go RT Go trials	−0.071	−0.025	−0.037	−0.068	−0.136	−0.157	−0.035
Simon effect 100 ms	0.231	0.188	0.088	0.016	0.080	−0.042	−0.056
Simon effect 900 ms	0.205	0.420^**^	−0.175	0.162	0.164	−0.035	−0.037

N = 135 for Conditioned Inhibition ratio; N = 50 for executive inhibition measures;

* *p < 0.05*,

** *p < 0.01*,

*** *p = 0.001 (FDR adjusted; Benjamini and Hochberg, 1995); CI, Conditioned Inhibition; ACC, accuracy; RT, reaction time*.

## Discussion

The purpose of the present study was to examine the association of different aspects of ADHD- and TS-like behaviors in the general population and the extent to which they are linked to different types of response inhibition deficits. The current study allowed us to assess the unique effects of the different aspects of ADHD- and TS-like behaviors (including OCBs) in automatic and effortful response inhibition deficits. For comparability, we used the same CI task used earlier in studies of clinical ADHD and TS (Kantini et al., 2011a,b) to assess automatic attentional response inhibition in a large sample of participants from the general population. To tap into various executive response inhibition processes, we used a standard Stroop task that requires the active suppression of a competing response to measure interference control and the Go/No-Go task which assesses the inhibition of a pre-potent motor response to measure behavioral inhibition. We also included the Simon task, (which has not previously been used in this context) to assess response selection interference between two independent processing paths—in this case spatial and perceptual processing (Simon and Rudell, 1967; Simon, 1969).

### Associations between subtypes of ADHD and TS

The factor analysis of the TS behavioral items yielded a structure readily interpretable in relation to the phenomenology of the condition, and these scales showed differential associations. The two factors distinguished between phonic and motor tics associated behaviors—in line with the distinction highlighted in TS symptomatology and diagnosis (Leckman et al., 2006). Keeping the OCBs separate allowed us to examine pure TS-like behaviors and co-occurring OCBs characteristic of TS as distinct variables. The associations between TS- and ADHD-like behaviors are consistent with the comorbidity of these conditions clinically. The zero-order correlations showed significant positive associations of all ASRS scales with overall, phonic and motor TS-like behaviors; and only ASRS-hyperactivity/impulsivity was related to increased OCBs. However, the regression analyses assessing the unique contribution of each ASRS subscale highlight a distinct pattern. Taking the covariation amongst the two scales into account, ASRS-hyperactivity/impulsivity was uniquely associated with increased phonic, motor and obsessive-compulsive TS-like behaviors, perhaps highlighting the role of impulsivity in the inability to inhibit unwanted phonic and motor tics as well as repetitive OCBs (Abramovitch and Schweiger, 2009). On the other hand, ASRS-inattention was uniquely associated with reduced OCBs linked to TS, but not to pure TS-like behaviors. This suggests that OCBs may demand high levels of attention paid to their repetitiveness and compulsiveness. Indeed, these opposite associations of hyperactive/impulsive and inattentive ADHD-like behaviors with OCBs may reflect the paradox of OCD-ADHD comorbidity—despite being very different disorders on opposite ends of a compulsive-impulsive continuum, and comprising different symptomatology and neurobiology (Hollander, 2005; Abramovitch et al., 2012).

According to the executive overload model of OCD (Abramovitch et al., 2012), continuous excessive attention paid toward controlling behavior leads to an overflow of obsessive thoughts, which causes an executive overload resulting in neurological impairment such as executive response inhibition deficits. This in turn leads to further increased attention and preoccupation with controlling those automatic processes, which in turn leads to further executive overload. Indeed, because of this vicious cycle, individuals with OCD tend to feel out of control (Abramovitch et al., 2012). Moreover, the opposite associations of hyperactivity/impulsivity and inattention ADHD-like behaviors with TS-related OCBs in the current study, may be in line with previous findings that increased obsessive-compulsive symptoms within a clinical ADHD sample were associated with better executive function performance—thought to reflect increased organization and attention to details in those ADHD individuals with OCD-like symptoms compared to those without (Abramovitch and Schweiger, 2009). However, given the low reliability of the (three-item) tailored TS-OCB scale, these findings have to be treated as preliminary, and should be replicated using more specifically designed psychiatric rating scales for OCD. Nevertheless, the overall findings suggests that the clinical comorbidity between ADHD and TS may be driven by the hyperactive/impulsive type, and that indeed pure Attention Deficit Disorder (ADD) may be less likely to show “comorbidity” with TS, which warrants further examination in clinical samples. Moreover, the opposite direction of effects of the two ASRS subscales led to suppressor effects for overall ASRS, which was not associated with TS-OCB. Taken together, these findings highlight the importance of examining the sub-types of ADHD (and indeed TS) separately in clinical populations, and may help to explain mixed or inconsistent findings in some previously reported studies.

### Yawning as a behavioral model of TS

Analysis of the Yawning scale yielded a three factor solution distinguishing between active, inactive and stress induced yawning. The Yawning scales were significantly positively correlated with pure (and overall) TS-like behaviors, and YWN-inactive was also associated with compulsive TS behaviors. The Yawning scales also showed some associations with ADHD-like behaviors, whereby YWN-activity (and overall) was significantly positively associated with all ASRS scales, and YWN-inactivity with hyperactive/impulsive (and overall) ASRS. However, YWN-stress was not linked to ASRS. The regression analyses for the unique contribution of the TS and ASRS scales (thus accounting for covariation amongst those) showed that only the three TS-like behaviors remained significantly associated with yawning during inactivity, and only motor TS-like behaviors with yawning during stressful situations involving self-presentation/awareness. The ASRS scales were not linked to these two yawning scales. In contrast, only phonic (but not motor or OCB) TS-like behaviors and inattentive (but not hyperactive/impulsive) ASRS were positively associated with yawning during activity involving concentration. In general, the findings support the notion that excessive yawning is associated with TS (Dalsgaard et al., 2001; Walusinski et al., 2010), but suggest that the behavioral yawning model may be more specific to TS in the context of relaxation (for all TS behaviors) or during self-awareness and stress (specifically for motor tics). Secondly, phonic tics, characterized by involuntary sounds produced by moving air through the nose, mouth, or throat, and underlying the same muscle groups as yawning itself (Leckman et al., 2006), are linked to yawning across different everyday situational contexts (inactive and active). Given the role of premonitory sensations in tic-generating S-S associations (Robertson, 2000), this suggests that yawning in those situations may trigger more common phonic tics. Yawning during stressful situations involving greater self-awareness, however, may be more likely to trigger motor tics, which also involve different muscle groups (Leckman et al., 2006). Thus, taking the situational context of premonitory sensations associated with different symptom expression into account may be useful in studying homogenous subgroups of TS (Prado et al., 2008). However, the finding that ASRS-inattention was associated with yawning during activity suggests that the behavioral yawning model may also be useful for ADD, and warrants further investigation in clinical samples. Given that yawning is thought to increase arousal (Walusinski et al., 2010), it may well be a functional response to increase attention during situations where concentration is required.

### Inhibitory control deficits in ADHD and TS

Superficially, ADHD and TS present as quite different conditions clinically. However, behavioral disinhibition is a feature of these disorders (Ozonoff et al., 1998; Sheppard et al., 1999; Thibault et al., 2008), and they rely on the normal functioning of the dopamine system (Gilbert et al., 2004). Surprisingly then, relatively few studies have directly compared participants with TS and ADHD, and those that have, generally studied comorbid groups rather than comparing separate groups. Taken together, clinical studies suggest that individuals with confirmed diagnoses of ADHD and/or TS do not reliably show consistent impairments across the range of tests used to measure behavioral and cognitive inhibition. That inhibition deficits are not reliably demonstrated under experimental conditions is at face value paradoxical in that (broadly defined) deficient inhibitory processes have been highlighted in both ADHD and TS. One possibility is that some of the earlier clinical studies reporting negative results were underpowered because of the difficulty in recruiting participants with ADHD in the absence of TS (or other comorbidities) and vice versa. Nigg (2000) highlighted that control of comorbid symptoms should be more routinely applied, and indeed, simultaneous comparison of different symptoms on several inhibition tasks is needed. Therefore, though the main aim of the study was to examine CI in relation to ADHD- and TS-like behaviors, we also compared different aspects of inhibitory control by incorporating standard executive control tasks in a subsample, in addition to the automatic inhibition task. Controlling for associations (“comorbidity”) with each other, significant relationships of ADHD and/or TS-like behaviors as measured by questionnaires with performance on the experimental inhibitory learning tasks were nonetheless limited in number.

From a learning perspective, S-R habit learning has been typically used in clinical studies, however, fewer studies have looked at S-S learning and the role of automatic inhibition in relation to TS and ADHD. In general, these studies have found some impairments in comorbid TS and OCD (e.g., negative priming; Fox, 1995; Ozonoff et al., 1998), though not in cases of pure TS or ADHD (Swerdlow et al., 1996; Ozonoff et al., 1998; Nigg et al., 2002; Yuen et al., 2005). Moreover, only two studies have specifically examined CI in ADHD (Kantini et al., 2011a) and TS in the absence of co-morbid ADHD (Kantini et al., 2011b), and both showed that performance was dependent on medication. However, there are no studies that have looked at these associations in the general population and/or taking subtypes of the disorders into account. This is important as automatic inhibition deficits may be more prominent in ADHD inattention (Aman et al., 1998) and obsessive-compulsive TS behaviors. Therefore, we included a measure of CI as a translational model developed from the animal learning designs and adapted for use with human participants (e.g., Williams et al., 1994; O'Boyle and Bouton, 1996; Karazinov and Boakes, 2004; Migo et al., 2006; He et al., 2013). However, similar to the lack of difference in diagnosed groups—other than in relation to medication (Kantini et al., 2011a,b)—there was no indication that individual differences in the different aspects of ADHD- or TS-like behaviors were associated with performance in the Pavlovian inhibitory learning task. Thus, this study adds to the previous studies that found no automatic inhibition deficits in TS or ADHD in clinical samples using the same paradigm.

Regarding effortful executive inhibition, ASRS-hyperactivity/impulsivity but not inattention was related to interference control deficits in the Simon task but only at longer not shorter waiting intervals. Increased reaction times are typically indicative of slower processing and/or more errors when perceptual stimulus-response associations are incongruent with spatial locations. This suggests executive inhibitory deficits in the hyperactive/impulsive subtype when simultaneous processing of independent pathways are involved, and particularly when it becomes more effortful due to longer time intervals. Conversely, ASRS-inattention but not hyperactivity/impulsivity was associated with *reduced* reaction times in the Stroop task for both incongruent and congruent trials, thus suggesting generally faster performance; or motivation deficits though this is less likely given that accuracy was not adversely affected. Moreover, ASRS-inattention was also selectively associated with reduced Stroop interference, indicating if anything, enhanced interference control for this subtype. Thus, executive inhibitory processing deficits seen in clinical combined or inattentive ADHD subtypes (Willcutt et al., 2005) were not replicated in this sample from the general population. At the same time, overall TS was associated with *increased* Stroop reaction times for congruent trials, suggesting generally slower automatic processing of the stimuli, and greater accuracy, without any interference control deficits. Indeed, Channon et al. (2009) also observed slower response times in TS compared to controls on several measures, particularly evident under conditions with greater inhibitory demands. However, we did not find a consistent pattern of slower reaction times across Stroop conditions or indeed across other tasks. Moreover, whilst phonic and motor TS were not associated with differences in task performance, TS-OCB was associated with increased accuracy in the Go/No-Go task. Clinically it has been shown that TS participants who are comorbid with OCD perform worse on tests of inhibition than those with a diagnosis restricted to TS (Ozonoff et al., 1998; Gilbert et al., 2004), and it has been suggested that studies which do not screen for comorbid illness may overestimate the level of impairment in TS (Nelson et al., 2011). Indeed, the current findings show that “pure” phonic or motor TS-like behaviors were unrelated to task performance, and the only dissociable relationship demonstrated with the TS behavior checklist was the lone correlation between TS-OCB and one parameter of inhibitory control performance. However, contrary to the above findings, obsessive-compulsive TS-like behaviors were associated with improved performance. Of course we should not put too much weight on a single correlation but in principle the TS-OCB scale could be of some use in identifying the relative role of an OCD-like disposition as a determinant of inhibitory control in other tasks in future studies. Taken together, TS in particular has long been viewed as a disorder of inhibition yet experimental studies with diagnosed participants typically find little deficit on tasks thought to involve inhibitory processes (Channon et al., 2003, 2006, 2009; Roessner et al., 2008). Moreover, an extensive series of studies provides compelling evidence consistent with enhanced cognitive control in (in some cases unmedicated) individuals with pure TS (Mueller et al., 2006; Jackson et al., 2007, 2011a; Vicario et al., 2010; Jung et al., 2014). These findings could relate to the development of functional compensation, triggered by the need to suppress tics, in the frontal lobes of TS sufferers (Serrien et al., 2005; Nelson et al., 2011). The present study lends support to the wider conclusion that TS-like behaviors do not necessarily occur in conjunction with deficits of inhibition as measured by standard human experimental procedures used to measure either effortful response inhibition or automatic associative inhibition.

### Limitations and further directions

The TS behavioral checklist should be seen as a preliminary measure: it has face validity given the inclusion of behaviors related to known DSM and ICD symptoms of TS. The split into three subtypes distinguishing between pure phonic and motor tics as well as OCBs makes theoretical sense. We felt that it was important to (i) add the OCBs to the scale, due to their known comorbidity with TS in clinical populations (Swerdlow, 2001; Rankins et al., 2005; Thibault et al., 2008), but (ii) keep those separate from the pure behaviors from the outset to be able to assess pure TS-like behaviors independently. Indeed, the correlations of OCBs with the phonic and motor behaviors were similar in strength, but significantly smaller compared to the correlation between the pure TS-like behaviors. Using this measure and the ASRS for ADHD-like behaviors we were able to examine subtype dependent associations between the two disorders, and with the behavioral yawning model and different aspects of inhibitory control. We identified unique associations of (i) phonic and motor TS-like behaviors with yawning during activity or stress, respectively, and (ii) OCBs with reduced inattentive aspects of ADHD and enhanced performance on the Go/No-Go task. However, the reduced item content and low reliability of the TS-OCB scale, and lack of further assessment of convergent validity of the whole checklist needs to be acknowledged. This limitation notwithstanding, the TS behavioral checklist developed in the present study—based on a dimensional approach to TS symptomatology—provides to our knowledge the first such measure, and can be used to further investigate TS-like behaviors and associated deficits in the general or clinical populations. Should the checklist withstand further investigation, the link between OCBs and the motor as opposed to phonic symptoms of TS should be further examined in clinical cases and may be useful for diagnostic refinement. For instance, future studies could examine to what extent OCBs are more likely in TS individuals who present motor tics rather than just the more common phonic tics in clinical populations. Alternatively, it is possible that phonic or motor tics present in TS individuals due to OCD comorbidity. Thus, assessing their unique associations may be useful in identifying more homogenous groups. The distinct associations we found in the current study point to the importance of addressing the subtypes of ADHD and TS in future research, ideally including clinical samples.

Finally, in order to examine both ADHD- and TS- like behaviors independently in a relatively large sample, as well as in relation to disorder sub-types, the present study used symptom-based measures of ADHD- and TS-like behaviors in normal participants who did not report any diagnosed condition. This could be seen as a limitation; however, given challenges posed by studies using clinical samples—due to the difficulty in recruiting participants who are medication free and without comorbidities, or samples sufficiently large to fully control for comorbid symptoms (Nigg, 2000)—the study of undiagnosed participants has its advantages. Thus, in line with the proposed continuity of symptom-related behaviors across the normal range, recent research has started investigating such associations in the normal population—at least in the case of ADHD (Kuntsi et al., 2005; Herrmann et al., 2009). Such a differential approach can both help to clarify the bases of clinical comorbidities and achieve more specificity regarding the proposed inhibition deficits seen in relation to these disorders. Nevertheless, it must be noted that although dimensional approaches have previously been adopted, future research will also need to examine the continuity of pathology-related cognitions and behaviors, to assess whether pathological and normal state differ just quantitatively or indeed qualitatively. In any case, caution is warranted when extending findings from the normal to clinical populations.

## Author contributions

Ebrahim Kantini administered the behavioral tests and questionnaire measures. Helen J. Cassaday contributed to the design and supervised data collection. Hannah L. R. Checkley conducted preliminary analyses under the supervision of Nadja Heym. Nadja Heym completed the final statistical analyses and wrote the manuscript. All authors contributed to the interpretation of the results, and the drafting and revision of the manuscript. All authors have approved the final manuscript.

### Conflict of interest statement

The authors declare that the research was conducted in the absence of any commercial or financial relationships that could be construed as a potential conflict of interest.
